# Transcriptomic Analysis of Tight Junction Proteins Demonstrates the Aberrant Expression and Function of Zona Occludens 2 (ZO-2) Protein in Stanford Type A Aortic Dissection

**DOI:** 10.3390/jpm13121697

**Published:** 2023-12-09

**Authors:** Dimitrios E. Magouliotis, Arian Arjomandi Rad, Antonios Kourliouros, Alessandro Viviano, Marinos Koulouroudias, Mohammad Yousuf Salmasi, Alexandros Briasoulis, Filippos Triposkiadis, John Skoularigis, Thanos Athanasiou

**Affiliations:** 1 Unit of Quality Improvement, Department of Cardiothoracic Surgery, University of Thessaly, 41110 Biopolis, Greece; 2 Department of Surgery and Cancer, Imperial College London, St Mary’s Hospital, London W2 1NY, UK; arian.arjomandi-rad16@imperial.ac.uk (A.A.R.); mybsalmasi86@gmail.com (M.Y.S.); t.athanasiou@imperial.ac.uk (T.A.); 3 Department of Cardiothoracic Surgery, Oxford University Hospitals, Oxford OX3 9DU, UK; antonios.kourliouros@ouh.nhs.uk; 4 Department of Cardiac Surgery, Hammersmith Hospital, Imperial College Healthcare NHS Trust, London W2 1NY, UK; alessandro.viviano@nhs.net; 5 Department of Cardiac Surgery, Nottingham University Hospitals NHS Trust, Nottingham NG5 1PB, UK; marinosk@doctors.org.uk; 6 Department of Therapeutics, Faculty of Medicine, National and Kapodistrian University of Athens, 10679 Athens, Greece; alexbriasoulis@gmail.com; 7 School of Medicine, European University Cyprus, Nicosia 2404, Cyprus; ftriposkiadis@gmail.com; 8 Department of Cardiology, University of Thessaly, Biopolis, 41110 Larissa, Greece; iskoular@gmail.com

**Keywords:** thoracic aortic dissection, biomarker, zona occludens 2, ZO-2, TJP2, miRNA

## Abstract

Objective: Thoracic aortic aneurysm dissection (TAAD) represents a cardiac surgery emergency characterized by the disrupted integrity of the aortic wall and is associated with poor prognosis. In this context, the identification of biomarkers implicated in the pathobiology of TAAD is crucial. Our aim in the present original in silico study is to assess the differential gene expression profile of the tight junction proteins (TJPs) in patients with TAAD and to propose novel biomarkers for the diagnosis and prognosis of this disease. Methods: We implemented bioinformatics methodology in order to construct the gene network of the TJPs family, identify the differentially expressed genes (DEGs) in pathologic aortic tissue excised from patients with TAAD as compared to healthy aortic tissue, and assess the related biological functions and the associated miRNA families. Results: Data regarding the transcriptomic profile of selected genes were retrieved and incorporated from three microarray datasets, including 23 TAAD and 20 healthy control samples. A total of 32 TJPs were assessed. The zona occludens 2 (ZO-2) protein encoded by the gene TJP2 was significantly under-expressed in patients with TAAD compared to the control group (*p* = 0.009). ZO-2 was associated with fair discrimination and calibration traits in predicting the TAAD presentation. CpG islands of ZO-2 were demonstrated. No important difference was found regarding ZO-2 expression between aneurysmal non-dissected and healthy control aortic tissue. Finally, we performed gene set enrichment analysis (GSEA) and uncovered the major biological functions and miRNA families (hsa-miR-155-5p, hsa-miR-1-3p, hsa-miR-2118-5p, hsa-miR-4691-3p, and hsa-miR-1229-3p) relevant to ZO-2. Conclusions: These outcomes demonstrated the important role of ZO-2 in the pathobiology of TAAD.

## 1. Introduction

Thoracic aortic aneurysms (TAAs) represent a pathologic entity that is mainly characterized by a distorted cellular and extracellular matrix architecture and composition in the aortic wall [1]. These distortions have been associated with certain etiologic factors that directly implicate the different stages of TAA pathogenesis, like genetic and inheriting syndromes, the presence of a bicuspid aortic valve, and idiopathic TAAs. In the same context, thoracic aortic aneurysm dissection (TAAD) represents a life-threatening cardiovascular emergency, and the early diagnosis and effective treatment still remain challenging. To date, and despite the extended research [2], the comprehension of TAAD pathogenesis has primarily focused on the degradation of elastic fibers, along with medial degeneration; however, there is only limited evidence on the function and status of endothelial cells (ECs) in TAAD. Tight junctions (TJs) are crucial pillars in sustaining endothelial integrity between ECs. There is growing evidence [3,4,5,6] highlighting the important role of smooth muscle cells (SMCs) in TAA, while also revealing the differences in the expression of specific SMC genes compared with the healthy aortic wall. However, these studies [2,3,4,5,6] have not assessed either the functional properties of the endothelial cells nor the mechanisms underlying the impaired integrity of the intercellular junctions. Consequently, the potential role of endothelial cells, as expressed through the distorted function of their junctions during the different stages of aneurysm and dissection progression still remains largely unknown today.

If we examine the role of endothelial cells in more detail, they seem to have a direct implication on SMC differentiation and phenotype [7,8,9]. In fact, a major role of the vascular SMCs is to preserve vascular tone while regulating the blood pressure through either constriction or relaxation. To succeed in these challenging functions, SMCs express several genes that implicate in intercellular junction proteins, thus providing the appropriate machinery for this response [9]. In this context, it is the tightness of these intercellular junctions that regulates both vascular tone and vascular permeability [10]. Nonetheless, there are certain pathological conditions in which a disruption in those genes’ expression is present, thus making the vascular wall significantly immature and leaky. In spite of their significant effect on the integrity of vascular tone and permeability, there is still only limited evidence available regarding their altered expression profile in the context of Stanford type A TAAD. In fact, to the best of our knowledge there is only one recent study investigating the role of tight junction proteins (TJPs) in abdominal, and not thoracic, aortic dissections [11]. According to this study, there is an important deregulation of certain TJPs in pathologic abdominal aortas, thus affecting the integrity of the endothelial cell barrier [11].

Taking into consideration the previously reported observations, the goal of the present study was to, for the first time, assess our hypothesis that TJPs function is deranged in patients with Stanford type A aortic dissection. In this context, we used in silico methodology [12] to assess the transcriptomic profile of the TJPs in this pathologic condition to identify potential biomarkers at the epigenetic level.

## 2. Materials and Methods

### 2.1. Assessment of the Transcriptomic Profile of the TJPs in Stanford Type A TAAD

We employed the PubMed Gene Expression Omnibus (GEO) database (https://www.ncbi.nlm.nih.gov/gds, accessed on 1 April 2023) to evaluate the expression profile of TJPs in patients with Stanford type A TAAD compared with healthy controls. PubMed GEO is an electronic dataset bank of publicly available gene expression curated data. We thoroughly hand-searched in PubMed GEO employing the following keywords: “thoracic aortic aneurysm dissection”, “taad”, and “aortic dissection” restricted to “Homo Sapiens” as the tissue donator species and Stanford type A classification. The search was performed during April 2023, by two independent researchers (DEM, AAR). The level of agreement between the reviewers was assessed using the kappa coefficient.

We identified the differentially expressed genes (DEGs) using three TAAD microarray datasets (GSE98770, GSE52093, GSE153434) [13,14,15], incorporating a total of 43 samples (23 TAAD samples and 20 healthy control samples). Pathologic tissue biopsies for RNA and histological analyses were obtained from pathologic dissected aortas of patients undergoing ascending aortic replacement surgery. Control tissue samples of normal non-dissected walls of the thoracic aorta were obtained from patients undergoing coronary artery bypass grafting surgery (CABG) without any aortic disease. The gene expression data were log-transformed and log Fold Change (logFC) was calculated. The null hypothesis proposed that the transcriptomic profile as compared between TAAD and normal aortic tissue samples was similar. Gene over-expression or under-expression was considered significant when the *p* value was lower than 0.05.

### 2.2. Validation of DEGs as Prognostic Markers for Patients with TAAD

We evaluated both discrimination and calibration of each DEG. The ability of DEGs to separate those patients who presented TAAD from those who did not present was considered as discrimination, while the ability of DEGs to predict the presentation of TAAD in agreement with actual observed TAAD rates was considered as calibration. Discrimination was evaluated by producing receiver-operating characteristic (ROC) curves and by estimating the area under the ROC curve (AUC). The AUC was determined by estimating the 95% confidence intervals (95% CI) and compared using nonparametric paired tests using the methodology described by DeLong et al. [16]. Model discrimination was defined as poor, fair, and excellent when the AUC values corresponded to <0.70, 0.70–0.79, and 0.80–1.00, respectively. The calibration regarding each model was assessed by estimating the predicted TAAD presentation (expected) and then compared with the true TAAD incidence (observed). The observed/expected (O:E) ratio of 1 represents perfect accuracy, a ratio < 1 demonstrates overprediction of TAAD rate, and a ratio of >1 corresponds to underestimation. We further assessed calibration traits by employing the Hosmer–Lemeshow (H-L) goodness of fit test, defining a lack of fit as a *p* value ≤ 0.05 [17]. Finally, Chi-squared testing was used to compare the observed and expected outcomes of all patients.

### 2.3. Nucleotide Sequential Analysis (NSA) of DEGs and Construction of Their Interactome

The nucleotide sequences of the DEGs were extracted in FASTA format from the Ensembl database. Following that, we employed the EMBOSS_CpGplot tool [18] to demonstrate the putative CpG islands using the criteria by Takai and Jones [19]. CpG islands are DNA methylation regions located at the promoters that regulate gene expression profile through silencing of transcription of related genes. Bioinformatic analysis of the DEGs-related interactome was performed to assess potential related factors implicated in TAAD pathogenesis. The DEGs gene network was generated by employing the GeneMANIA platform (http://genemania.org/, accessed on 1 April 2023) [20]. The functions of the proteins associated with the identified genes were extracted from the portal GeneCards (http://www.genecards.org/, accessed on 1 April 2023), a database providing information on human genome.

### 2.4. Gene Set Enrichment Analysis (GSEA) Regarding the Biological Functions and Regulating miRNAs

The Gene Set Enrichment Analysis (GSEA) of Gene Ontologies (GO) was performed via STRING and DSigDB [21], available through the Enrichr tool [22,23]. Enrichr provides direct information regarding the DEG-associated biological functions, while it implements data from the miRTarBase regarding the regulating miRNAs [24]. MiRTarBase is an electronic database that contains more than three hundred and sixty thousand miRNA-target interactions (MTIs). The included MTIs are further validated experimentally by reporter assay, Western blot, microarray, and next-generation sequencing experiments [24]. The analyses were performed during May 2023. GSEA enables a deeper insight in the molecular, biological, genetical, and phenotypical functions and pathways associated with the genes of interest.

### 2.5. Evaluation of the Identified DEGs Expression Profile in Aneurysmal Aortic Tissue without Dissection

In order to assess the exact stage of disease progression at which the aberrant expression of previously identified DEGs begins, we analyzed their expression profile in tissue samples from dilated aortas without dissection and compared it with normal control aortic tissue. In this context, we retrieved microarrays data from one dataset in PubMed GEO (GSE26155) [6]. A total of 86 tissue samples were assessed (43 normal aortic tissue samples and 43 pathologic dilated aortic tissue samples).

### 2.6. Statistical Analysis

We performed all statistical analyses by employing the software GraphPad Prism 10.0.3 for Mac (GraphPad Software, San Diego, CA, USA). The type of distribution (normal versus non-normal distribution) of parameters was assessed through employing the D’Agostino and Pearson Omnibus normality test. In addition, the comparison of gene expression levels was performed with a two-tailed unpaired t-test for parametric data and Mann–Whitney U-test for nonparametric data. Furthermore, we corrected all *p* values for multiple comparisons by employing the two-stage, step-up False Discovery Rate (FDR) approach (Benjamini, Krieger, Yekutieli method) and by estimating the *Q* statistic (Desired *Q*: 1%). Correlations were further evaluated by estimating the Pearson or the Spearman’s rank (ρ) correlation coefficients for parametric or non-parametric data, respectively. The Area Under the Curve was determined by calculating the 95% confidence intervals (95% CI) and compared using nonparametric paired tests, as described by DeLong et al. [16]. We defined as poor, fair, and excellent model discrimination the AUC values of <0.70, 0.70–0.79, and 0.80–1.00, respectively. Calibration was evaluated using the Hosmer–Lemeshow (H-L) goodness of fit test, defining a lack of fit as a *p* value ≤ 0.05 [17]. Finally, we performed Deming regression analysis to assess the cause-and-effect relationships among DEGs. Differences were considered significant (rejection of the null hypothesis) with a *p* value lower than 0.05.

## 3. Results

### 3.1. Assessment of the Transcriptomic Profile of the TJPs in Stanford Type A TAAD

**Figure 1:**
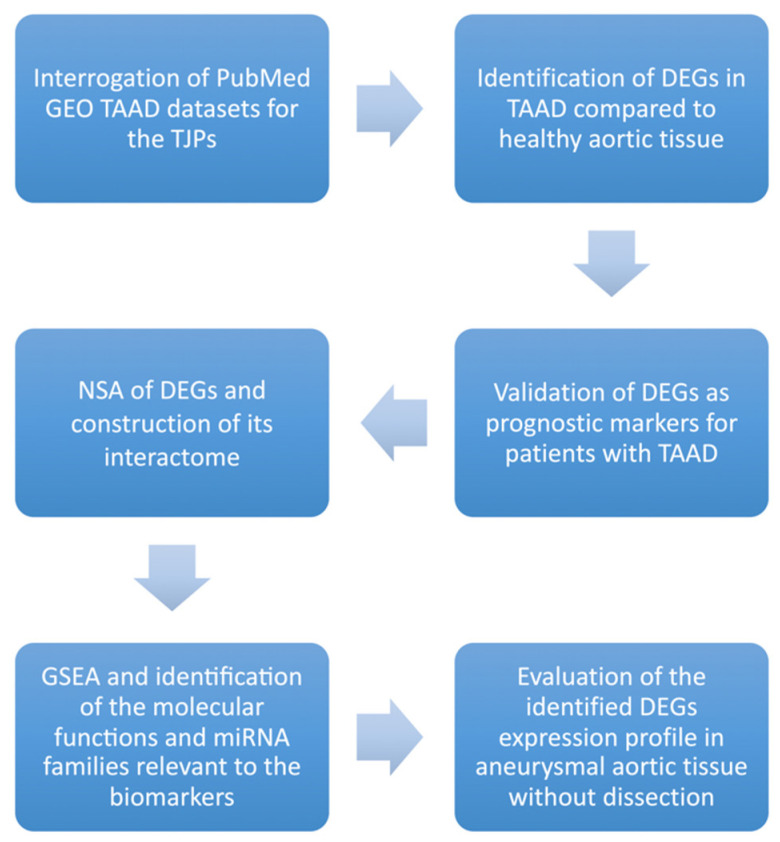
Trial flow of the current study. Abbreviations: TAAD: thoracic aortic aneurysm dissection; TJPs: tight junction proteins; DEGs: differentially expressed genes; NSA: nucleotide sequential analysis; GSEA: Gene Set Enrichment Analysis.

A trial flow of the current study is demonstrated in Figure 1. The list of TJPs that were assessed is demonstrated in Table 1. We started by interrogating the PubMed GEO database for datasets with aortic tissue samples from patients with thoracic aortic dissection to calculate the expression profile of the tight junction proteins. Secondly, we identified the differentially expressed genes in TAAD compared to healthy aortic tissue. The next step was to validate the DEGs as prognostic markers for patients with TAAD. In addition, we performed a nucleotide sequential analysis of the DEGs and we constructed their interactome. Moreover, we performed Gene Set Enrichment Analysis (GSEA) and identified the DEGs-associated molecular functions and miRNA families. Finally, we evaluated the expression profile of DEGs in aneurysmal aortic tissue without dissection. Although, our original purpose was to directly compare the expression profile of DEGs between aneurysmal aortic tissue with and without dissection, this comparison could not be performed due to the methodological heterogeneity among the different datasets.

**Figure 2:**
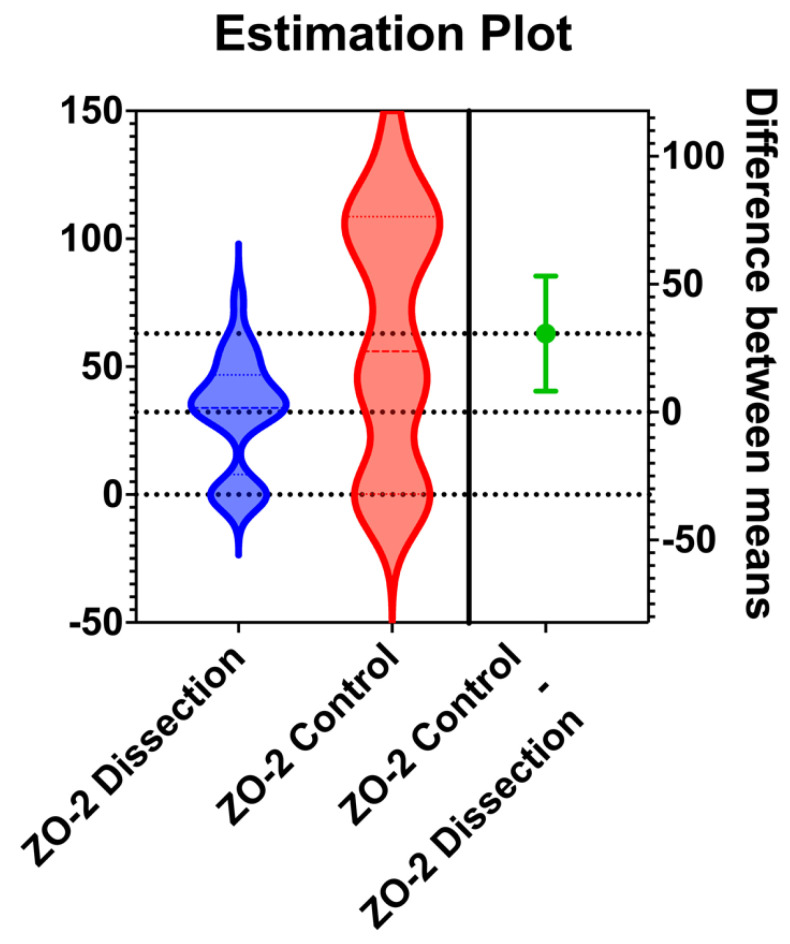
Estimation plot demonstrating the under-expression of ZO-2 protein in pathologic aortic tissue with dissection.

Three PubMed GEO TAAD microarray datasets were identified (GSE98770, GSE52093, GSE153434). There was a substantial level of agreement between the two reviewers as was defined by the Kappa coefficient estimation (Kappa = 0.642; 95% CI: 0.005, 1.000). The three datasets incorporated 43 samples (23 TAAD samples and 20 normal control samples). Out of the 32 TJPs, there has been sufficient PubMed GEO data on their gene expression level about 31 genes (96.9%). One DEG, ZO-2, was identified as presented in Figure 2. In fact, ZO-2 was under-expressed in aortic tissue excised from patients with TAAD (*p* = 0.009; logFC = 0.5). No significant difference was reported regarding the expression level of the other genes.

### 3.2. Validation of DEGs as Prognostic Markers for Patients with TAAD

**Figure 3:**
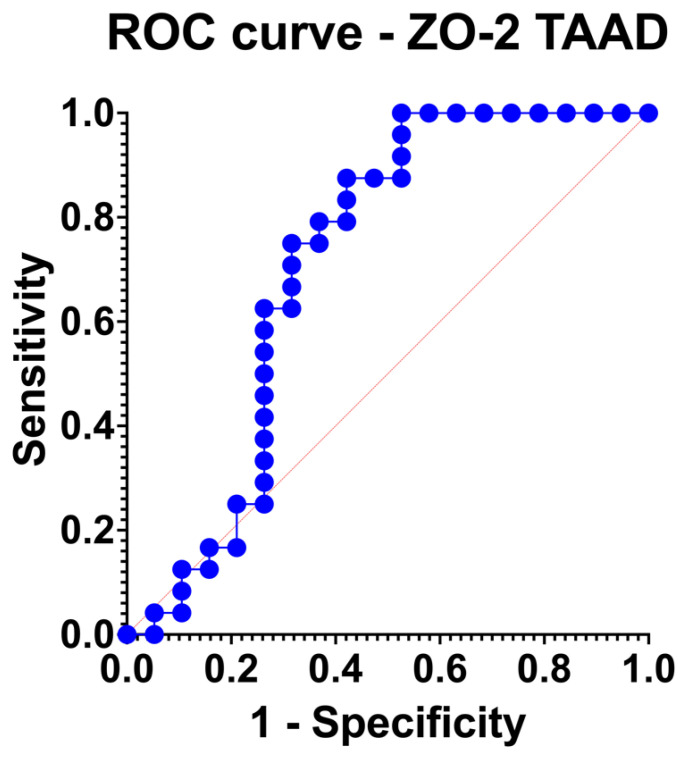
Receiver operating characteristic (ROC) curve demonstrating the discrimination traits of zona occludens 2 (ZO-2) protein expression profile to predict thoracic aortic aneurysm dissection.

ZO-2 was associated with a fair discrimination level (AUC: 0.71 [95% confidence intervals: 0.53, 0.89]; *p* = 0.02) (Figure 3). In addition, ZO-2 passed the goodness of fit test (H-L: 10.80; *p* = 0.21).

### 3.3. Nucleotide Sequential Analysis (NSA) of DEGs and Construction of Their Interactome

**Figure 4:**
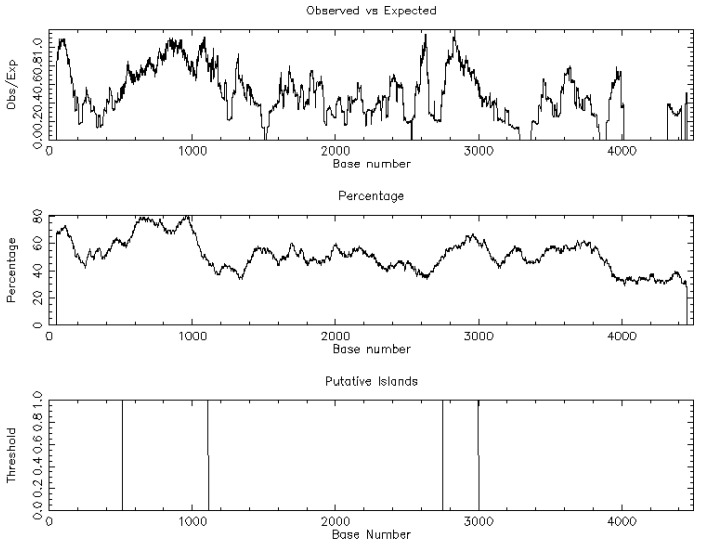
Nucleotide sequential analysis of zona occludens 2 (ZO-2) protein demonstrating two CpG islands.

The nucleotide sequential analysis (NSA) demonstrated two CpG islands [length: 600 (513, 1112); length: 247 (2754, 3000)] related to ZO-2 (Figure 4), using the following criteria: Observed/expected ratio > 0.60; percent C+ percent G > 50.00; length > 200.

**Figure 5:**
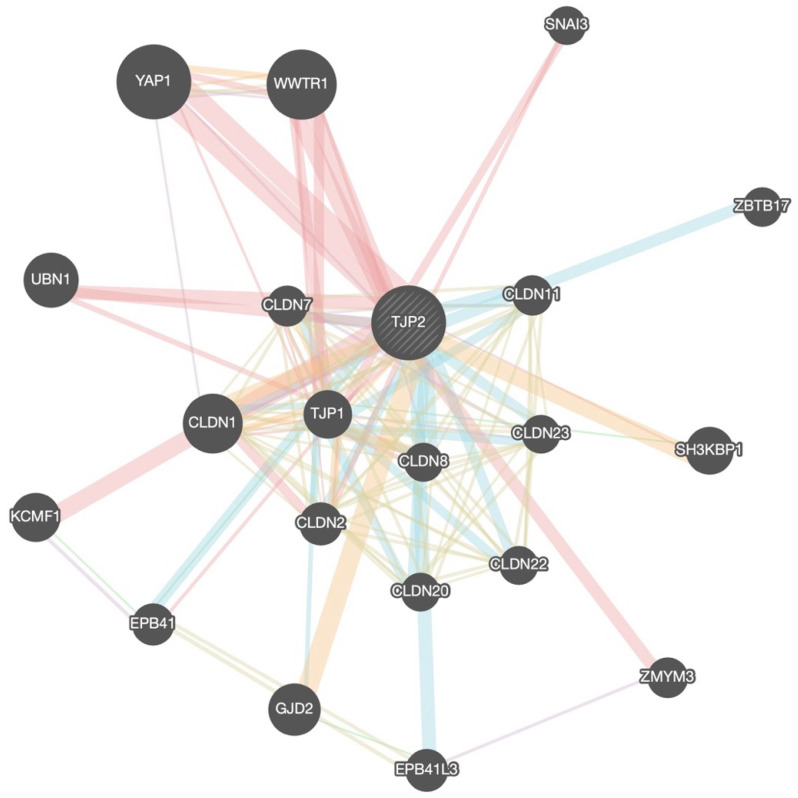
The gene network of the ZO-2 protein encoded by the differentially expressed gene TJP2.

The members of the ZO-2 gene network that were extracted from the GeneMania platform are demonstrated in Figure 5. A total of 20 interacting proteins were revealed through the construction of the ZO-2 (encoded by TJP2 gene) interactome in homo sapiens.

### 3.4. Gene Set Enrichment Analysis (GSEA) Regarding the Biological Functions and Regulating miRNAs

ZO-2 underwent GSEA (Table 2). The top five enriched Gene Ontology terms for biological functions are presented in Table 2, along with the miRNAs that regulate the eight DEGs. Regulation of cell–cell junction organization and assembly, (hemi)desmosome organization and assembly, and cell migration, along with the integrin-mediated signaling pathway, represented the most important biological functions associated with the ZO-2 gene. Finally, the GSEA demonstrated that the members of the hsa-miR-155-5p, hsa-miR-1-3p, hsa-miR-218-5p, hsa-miR-4691-3p, and hsa-miR-1229-3p miRNA families were significant regulators of ZO-2.

### 3.5. Evaluation of the Identified DEGs Expression Profile in Aneurysmal Aortic Tissue without Dissection

Finally, we evaluated the expression level of the ZO-2 protein in aneurysmal thoracic aortic tissue. According to our outcomes, there was no significant difference between the aneurysmal and the healthy control aortic tissue samples (*p* = 0.80), thus suggesting that its function is deregulated at a later stage of the disease progression.

## 4. Discussion

The present study employed in silico techniques to evaluate the gene expression profile of the TJPs interactome in the context of TAAD pathobiology by implementing data provided by three TAAD microarray datasets, thus providing our analyses with an enhanced accuracy at the molecular level. The bioinformatics methodology is of great value to identify potential biomarkers and treatment targets, functioning as a filter to accurately guide translational research in a timely and cost-effective manner [20]. In this context, a total of 43 samples were included and analyzed in the current study. In addition, we demonstrated the nucleotide CpG islands and generated the interactome of the ZO-2 gene through which we uncovered 19 additional related genes. The outcomes provided in the current study should be further investigated so as to fully unveil the role of ZO-2 protein in the biology of TAAD, along with its potential use as a diagnostic/prognostic marker, or even a treatment target. 

The aortic wall is made of the following three distinct tissue layers: the intima, the media, and the adventitia, each one with its characteristic components. In fact, the intima is composed of a single layer of endothelial cells (ECs). In the same context, the media is mainly composed of SMCs, while in the adventitia, the fibroblasts are predominant. Although other components, such as mesenchymal stem cells, pericytes, and immune cells also play a crucial role in aortic wall integrity, SMCs have been considered the most important cell type in the pathogenesis of TAAD [3,4,5,6]. However, in the intima, which is composed of the thin layer of ECs, adherens, and tight junctions proteins or cytoplasmic actin-binding proteins [e.g., zona occludens (ZO) proteins] represent major biological pillars of the wall integrity. In a previous study, our team demonstrated the role of adherens junction proteins in the pathobiology of TAAs [25]. According to that study [25], a molecular signature of eight genes (CDH5; Calcitonin Receptor-Like Receptor-Kinase Insert Domain Receptor–KDR, CALCRL; Activin A Receptor-Like Type 1-ACVRL1, Purinergic Receptor P2X 4-P2RX4, Protein Tyrosine Phosphatase Receptor Type J-PTPRJ, Tryptophanyl-TRNA Synthetase 1–WARS, and Junction Plakoglobin-JUP) were identified as potential biomarkers associated with thoracic aortic aneurysms. In addition, PTPRJ demonstrated excellent discrimination and calibration traits in predicting the presentation of TAA. In the same context, Yang et al. were the first to investigate the biological role of TJPs in dissected aortic tissue and to highlight a potential role of the aberrant expression of TJPs in the etiology of TAAD [26]. Following, a different methodological path, our also study demonstrates the role of the cytoplasmic actin-binding proteins and, specifically, ZO-2, in the pathogenesis of TAAD.

Tight junction protein ZO-2 in humans is encoded by the TJP2 gene [27]. TJPs belong to a family of homologs of the membrane-associated guanylate kinase (MAGUK) and are implicated in the organization and function of epithelial and endothelial intercellular junctions. Herein, we demonstrate the under-expression of the ZO-2 protein in dissected aortic tissue. Nonetheless, according to our findings, the expression of ZO-2 is not significantly deregulated in aneurysmal non-dissected aortic tissue, thus proposing that it is implicated in the later stages in the progress of the disease and is potentially associated with a worse prognosis. In the same context, we found that ZO-2 is associated with fair discrimination traits in predicting dissection of the thoracic aortic wall. Our hypothesis-driven outcomes suggest that patients with a known history of TAA with a severe dysregulation of ZO-2 might present important disruptions of tight junctions between endothelial cells of the intima, thus leading to TAAD and a worse prognosis. This valuable information should be further investigated at the translational level to enhance our level of knowledge regarding the TAAD biology and our diagnostic/treatment options.

In the present study, we also identified the associated miRNA families that regulate the functions of the ZO-2 protein. In fact, through GSEA, we demonstrated that the hsa-miR-155-5p, hsa-miR-1-3p, hsa-miR-218-5p, hsa-miR-4691-3p, and hsa-miR-1229-3p miRNA families are important regulators of ZO-2. To the best of our knowledge, this is the first evidence suggesting that these miRNA families are implicated in the pathogenesis of aortic aneurysm and dissection. Previously, there has been evidence in the literature showing the implication of hsa-miR-155-5p, hsa-miR-1-3p, and hsa-miR-218-5p in endothelial dysfunction, pulmonary hypertension, and atrial fibrillation [28,29,30]. In addition, we demonstrated the primary biological functions related to the ZO-2 protein. These were associated with the regulation of cell–cell junction organization and assembly, (hemi)desmosome organization and assembly, cell migration, along with integrin-mediated signaling pathway. 

The certain study is associated with certain limitations. These are (1) the relatively small number of tissue samples, (2) the lack of multivariate analyses due to the small number of samples, and (3) the lack of mutation/alteration data that provide a stronger level of evidence compared to gene expression data. In addition, the technical and methodological variability among the datasets, along with the differences in clinical characteristics among the included patients represent another limitation. Given the lack of access to raw data from other studies, along with the small number of the included samples, we are limited to presenting our outcomes without further validation. Nonetheless, the study of Yang et al. [26] demonstrates outcomes that are in accordance with our findings. Finally, despite our original aim to directly compare the samples excised from aneurysmal non-dissected and dissected thoracic aortas, we could not perform such a direct comparison due to the different methodologies employed in dissection and non-dissection datasets, thus posing a certain bias. 

On the other hand, the strengths of the current study are (1) the clear protocol, (2) the investigation of the DEGs expression profile in two different stages of the disease (TAA without dissection and TAAD), and (3) the performance of GSEA, which indicated the biological functions and miRNA families related to ZO-2.

## 5. Conclusions

In the present study, we demonstrated the implication of deregulated expression and function of ZO-2 protein, encoded by the gene TJP2, in the pathogenesis and progression of TAAD. In addition, herein, we unveiled the properties of ZO-2 protein and demonstrated that ZO-2 deregulation is associated with the stage of disease progression from aneurysm to dissection, along with its fair discrimination and calibration traits in predicting TAAD presentation. Moreover, according to our outcomes, there was no significant difference in the ZO-2 expression profile between the aneurysmal and the healthy control aortic tissue samples (*p* = 0.80), thus suggesting that its function is deregulated at a later stage of the disease progression. We also investigated the nucleotide sequential properties of ZO-2, along with the predicted biological functions and relevant miRNA families. Given the bioinformatic nature of the current study, future translational research should further and fully unveil the potential benefit derived from our outcomes regarding the TAAD diagnosis and treatment.

## Figures and Tables

**Table 1:** The list of tight junction proteins (TJPs) that were assessed in the current study.

Gene Symbol	Gene Description
OCLN	Occludin
CLDN1	Claudin 1
CLDN2	Claudin 2
CLDN3	Claudin 3
CLDN4	Claudin 4
CLDN5	Claudin 5
CLDN6	Claudin 6
CLDN7	Claudin 7
CLDN8	Claudin 8
CLDN9	Claudin 9
CLDN10	Claudin 10
CLDN11	Claudin 11
CLDN12	Claudin 12
CLDN14	Claudin 14
CLDN15	Claudin 15
CLDN16	Claudin 16
CLDN17	Claudin 17
CLDN18	Claudin 18
CLDN19	Claudin 19
CLDN20	Claudin 20
CLDN22	Claudin 22
CLDN23	Claudin 23
CLDN24	Claudin 24
ZO-1	Zona Occludens 1
ZO-2	Zona Occludens 2
ZO-3	Zona Occludens 3
MAGI-1	Membrane-Associated Guanylate Kinase, WW And PDZ Domain Containing 1
MAGI-2	Membrane-Associated Guanylate Kinase, WW And PDZ Domain Containing 2
MAGI-3	Membrane-Associated Guanylate Kinase, WW And PDZ Domain Containing 3
PAR-3	Par-3 Family Cell Polarity Regulator
PAR-6	Par-6 Family Cell Polarity Regulator

**Table 2:** Enrichment analysis of gene ontologies (GO) for the prognostic factors. Top five relevant biological functions and regulating miRNA families are demonstrated.

	ID	Name	Adjusted *p* Value
1	GO:0045216	Cell–cell junction organization	<0.001
2	GO:0002934	Desmosome organization	<0.001
3	GO:0007043	Cell–cell junction assembly	<0.001
4	GO:0031581	Hemidesmosome assembly	<0.001
5	GO:0030334	Regulation of cell migration	<0.001
**Regulating miRNA Families**			
	**Name**		**Adjusted *p* Value**
1	hsa-miR-155-5p		0.003
2	hsa-miR-1-3p		0.037
3	hsa-miR-2118-5p		0.104
4	hsa-miR-4691-3p		0.142
5	hsa-miR-1229-3p		0.181

## Data Availability

The data supporting the article are available in the PubMed GEO at https://www.ncbi.nlm.nih.gov/gds, reference numbers GSE98770, GSE52093, GSE153434, and GSE26155.
